# The Impact of Breast-Conserving Therapy and Radiotherapy on Respiratory Parameters and Quality of Life in Women with Breast Cancer in Terms of Rehabilitation

**DOI:** 10.3390/jcm15041593

**Published:** 2026-02-18

**Authors:** Bartosz Mroczkowski, Paulina Okrzymowska, Krystyna Rozek-Piechura

**Affiliations:** 1 Lower Silesian Oncology, Pulmonology and Hematology Center, 53-413 Wroclaw, Poland; bartosz.mroczkowski@dcopih.pl; 2 Department of Physiotherapy in Internal Medicine and Oncology, University of Health and Sport Sciences, 51-612 Wroclaw, Poland; krystyna.rozek-piechura@awf.wroc.pl

**Keywords:** oncology, adjuvant therapy, postoperative care, physiotherapy, spirometry, inspiratory muscle training, WHOQOL-Bref

## Abstract

**Objectives**: The aim of the study was to assess the impact of oncological treatment including surgery and radiotherapy on the respiratory function and quality of life of women treated for breast cancer, considering the effects of physiotherapy and additional inspiratory muscle training. **Methods**: A quantitative, repeated-measures study included 26 women (aged 30–69) with breast cancer who had undergone breast-conserving surgery and radiotherapy, randomly assigned to an IMT group or a sham IMT group. The following tests were performed on each patient: respiratory function, respiratory muscle strength, the WHOQOL-Bref questionnaire. The tests were performed five times as follows: before surgery, after surgery (4–6 days), before the start of radiotherapy (4–5 weeks after surgery), after the end of radiotherapy, and follow-up 4 weeks after the end of radiotherapy. Group I-IMT: patients underwent physiotherapy according to hospital rehabilitation standards and inspiratory muscle strength training at 15–60% PI_max_. Group II-sham-IMT: patients underwent physiotherapy according to hospital rehabilitation standards and inspiratory muscle strength training at 15% PI_max_. **Results**: After the surgery, a reduction in all parameters was observed, which improved gradually and depending on the group after physiotherapy and inspiratory muscle training. The PI_max_ value decreased significantly after the procedure in both groups (*p* = 0.00), but its significant increase after 4 weeks and radiotherapy (*p* = 0.00) was noted only in the I-IMT group. The quality of life assessed by women (WHO1) was significantly higher (*p* = 0.009) only in the group using IMT training with a load of 60% PI_max_. **Conclusions**: Radical breast cancer treatment, including surgery and radiotherapy, significantly impairs respiratory function and quality of life in women, with the greatest deterioration observed after surgery. The use of prehabilitation and postoperative physiotherapy reduces the adverse effects of radical treatment, while additional inspiratory muscle training supports the improvement of respiratory function and the subjective assessment of quality of life in patients.

## 1. Introduction

Breast cancer is the leading cause of cancer deaths among women, but mortality rates have declined significantly over the past few decades. This reduction in mortality is primarily due to early detection through mammography screening and improved treatment of both early-stage and metastatic breast cancer [1,2,3].

Studies indicate that the decline in mortality is associated with the follwing three main factors: treatment of stage I-III disease (47%), treatment of metastatic disease (29%), and earlier detection through screening (25%) [1,2].

The WHO emphasizes that the growing burden of cancer, combined with limited access to cancer care in many countries, poses a serious public health challenge [4,5].

Current research indicates a significant reduction in breast cancer mortality when the disease is diagnosed at an early stage [6].

Currently, there is a trend in oncological surgery towards minimally invasive surgical procedures with possible reoperation after receiving histopathological test results. In the case of breast cancer, minimally invasive surgical treatment is used [7]. Surgical intervention reduces physical fitness, causes pain, and in some patients leads to psychological concerns about their health and prevents them from functioning properly in everyday life and engaging in physical activity. In addition, surgery results in damage to anatomical structures, which contributes to reduced mobility of the chest, thoracic and cervical spine, shoulder girdle, and upper limb [8,9,10]. A review of the literature on the assessment of the impact of radical treatment used in oncology confirms its negative effects on various body systems [8,9,11,12].

Cancer treatment carries several adverse consequences that lead to a deterioration in the patient’s health. The available literature indicates that breast-conserving surgery (BCS) combined with adjuvant radiotherapy provides local oncological control and improved overall survival rates comparable to mastectomy. Patients undergoing breast cancer treatment are significantly at risk for adverse respiratory effects and chronic fatigue syndrome. This is due to chemotherapy, thoracic surgery, and thoracic radiotherapy. Routinely administered adjuvant radiotherapy after BCS can cause deterioration of lung function. These changes are most evident in the first year after radiotherapy [11,13,14].

One of the most described cancer treatments that cause many negative effects in the human body are chemotherapy and radiotherapy [15]. According to the results of studies by Argüder et al., radiotherapy in relation to the respiratory system is considered one of the more invasive methods of treatment [11,16]. Radiotherapy causes conditions such as radiation pneumonitis, radiation fibrosis, bronchiolitis obliterans with organizing pneumonia, and eosinophilic pneumonia [11]. As Argüder et al. emphasize, radiotherapy causes various types of damage to the lungs and respiratory system, and the mechanism associated with radiation is still unclear and it is difficult to predict the risk factors for toxicity [11].

In addition to radical treatment methods such as chemotherapy, radiotherapy, and surgery, physiotherapy should be considered as an integral part of treatment. Persistent symptoms associated with adverse effects of treatment, such as pain and fatigue, can impair patients’ functional capacity and worsen their quality of life [13]. In this area, physiotherapy can play a significant role in supporting cancer treatment. This is confirmed by the results of a study conducted by Kalinowski et al. on 100 women after mastectomy, which confirmed that rehabilitation is an essential element of oncological treatment. In their study, the authors demonstrated that physical exercise and massage are the best rehabilitation methods after surgical treatment for breast cancer [17]. Rehabilitation reduces the adverse effects of therapy, including numbness at the surgical site, swelling of the upper limb, pain and limited mobility in the shoulder complexes, and weakening of the upper limb muscle strength on the operated side. Women undergoing oncological treatment for breast cancer should undergo rehabilitation prior to surgery. A literature review highlights the evaluation of currently used physiotherapy methods during oncological treatment among women treated for breast cancer. According to Przedborska et al., therapy for individuals struggling with cancer focuses primarily on improving physical activity by incorporating structured aerobic training into their daily routine [18]. The results of these authors’ studies indicate an increase in patients’ fitness, a reduction in pain, and symptoms resulting from hypokinesia. The authors also noted an improvement in patients’ well-being and self-esteem [18,19]. There are few reports in the available literature on the effectiveness of inspiratory muscle training in patients with breast cancer. Analyzing the available results of training in cardiovascular and respiratory diseases, it can be assumed that the use of inspiratory muscle training in patients with breast cancer will significantly improve the functionality of the respiratory, musculoskeletal, and cardiovascular systems [20,21]. The aim of the study was to assess the impact of oncological treatment including surgery and radiotherapy on the respiratory function and quality of life of women treated for breast cancer, considering the effects of physiotherapy and additional inspiratory muscle training.

## 2. Materials and Methods

The study was conducted at the Breast Unit, part of the Lower Silesian Center for Oncology, Pulmonology and Hematology in Wrocław. The project required a positive opinion from the Bioethics Committee of the Wrocław Medical University (no. KB-130/2019) and approval from the director of the Lower Silesian Oncology Center in Wrocław. The research data were obtained from March 2022 to July 2023.

This study was designed as a quantitative, repeated measures study to assess the impact of cancer treatment, including surgery and radiotherapy, on respiratory function and quality of life in women treated for breast cancer.

### 2.1. Participants

The study included 26 women with breast cancer, aged from 30 to 69, who had undergone breast-conserving surgery (BCT) and radiotherapy.

Written informed consent was obtained from the patients before the study began. Sample size estimation was performed using G*Power (G*Power 3.1, Düsseldorf, Germany) software version 3.1. Based on the adopted study design—two study groups (group I-IMT and group II-sham) and 5 repeated measures, the minimum required sample size was determined to be 26 participants.

Inclusion criteria: breast cancer, HER2-positive (HER2+), eligibility for preoperative neoadjuvant chemotherapy, BCT with sentinel lymph node aspiration and radiotherapy, informed and voluntary consent to participate in the research study, and age between 30 and 69 years.

Exclusion criteria included the presence of metastatic lesions, inflammatory breast cancer, infectious diseases, massive upper limb edema, acute circulatory system diseases, chronic respiratory diseases, and mental disorders that prevented patient contact and cooperation. Patients were also not allowed to actively smoke cigarettes.

### 2.2. Research Methods

All patients qualified for the study were randomly assigned to the following groups: I-IMT and II-sham-IMT. Randomization was performed using computer-generated random numbers (www.randomizer.com) URL (accessed on 21 March 2022). Once the inclusion criteria were met, each patient was assigned a random number, which designated her group assignment. The randomization process was overseen by the research team to ensure the integrity of group allocation, as follows:

Group I-IMT—patients underwent physiotherapy according to the hospital’s rehabilitation standard and strength training of the inspiratory muscles with appropriate load.

Group II-sham-IMT—this group underwent a sham procedure. The patients underwent physiotherapy according to the hospital’s rehabilitation standards and received an inspiratory muscle trainer, while the training load was set to the minimum range, determined by the technical limitations of the trainer—15% MIP [22].

**Figure 1:**
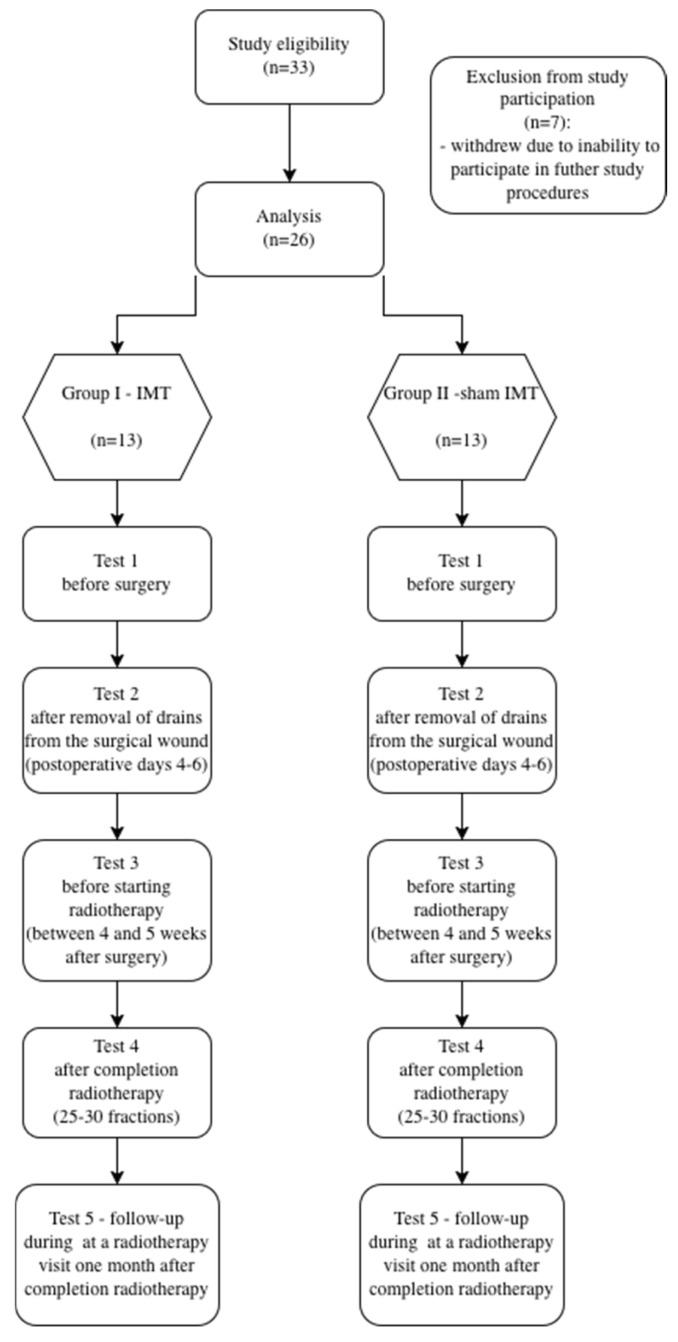
Design of the study.

The examinations of patients qualified for the project were carried out according to the following scheme (Figure 1):

Due to its specific nature, the quality-of-life assessment, which aims to evaluate the effects of cancer treatment (surgery, radiotherapy, physiotherapy procedures, and inspiratory muscle training) [23], was performed in study 1 (before surgery) and during study 5 (follow-up).

All patients underwent the following tests: measurement of somatic characteristics (age, height, and body weight), respiratory function, and inspiratory muscle strength using a Jaeager MasterScreen Pneumo (Jaeger Medical GmbH, CareFusion, Höchberg, Germany) spirometer with a pneumatic attachment.

Respiratory function was assessed using a Jaeager MasterScreen Pneumo spirometer (Jeager Medical GmbH, CareFusion, Höchberg, Germany). The following parameters were assessed during the examination: forced vital capacity [L]—Forced Vital Capacity (FVC); forced expiratory volume in one second [L]—Forced Expiratory Volume in one second (FEV_1_); and peak expiratory flow [L/s]—Peak Expiratory Flow (PEF).

The tests were conducted based on the current guidelines of the American Thoracic Society and European Respiratory Society (ATS/ERS), as well as the guidelines of the Polish Society of Pulmonary Diseases. In accordance with the procedure, 5 to 10 valid results were obtained. The normal difference was less than 5% or 5 cm H_2_O. The flow/volume curve was performed three times with an exhalation lasting at least 6 s [24,25,26]. The measurement of maximum inspiratory and expiratory pressure is a non-invasive method of clinical assessment of respiratory muscle strength, characterized by its simplicity, speed, and widespread approval [27,28].

The WHOQOL-Bref (World Health Organization Quality of Life Bref) questionnaire consists of 26 questions, the first two of which determine the respondent’s general perception of their quality of life and health. The following questions cover the following four domains: physical, psychological, social, and environmental. In each domain, it is possible to obtain between 4 and 20 points. When completing the questionnaire, the respondent can answer each question on a 5-point scale. The questionnaire also includes questions that are interpreted separately, i.e., individual overall perception of quality of life (WHO1) and individual overall perception of health (WHO2) [29].

### 2.3. In-Hospital Rehabilitation

At the Lower Silesian Oncology Center, in the Breast Unit, physiotherapy was conducted according to in-hospital standards and included the following: instruction by a physiotherapist who performed exercises with patients before the procedure, education about the disease, the procedure, daily living activities after treatment, and preventative care. Upon admission, patients received a set of informational materials describing postoperative exercises, an anti-edema wedge, and upper limb circumference measurements. The physical exercises were divided into three periods depending on the time elapsed since the procedure. During the first three days, the patient performed active, free-form upper limb exercises in a supine position (6 exercises, 30 repetitions each) and breathing exercises as a calming exercise. On days four to six, the patient performed the exercises from the first period combined with exercises in a sitting position using a cane (6 exercises, 30 repetitions each, and an additional four exercises with a cane, 15 repetitions each) and breathing exercises as a calming exercise. On subsequent days after the procedure, all exercises from the previous periods were performed, plus additional exercises in a standing position (an additional four exercises in a standing position, 15 repetitions each). The patient independently monitored her own anti-edema control. Participants were required to perform the exercises according to the materials and standards provided and to keep an exercise diary. The diary entries were reviewed, verified, and discussed with the participants by the physiotherapist responsible for the rehabilitation program. Completeness, adherence to the prescribed program, and any discrepancies reported by the patients were assessed.

### 2.4. Inspiratory Muscle Training

All patients enrolled in the project received instruction and a personal trainer for inspiratory muscle training using the Philips Threshold IMT device (Philips Respironics Threshold IMT Respiratory Trainer, Inc., Murrysville, PA, USA). Training was performed daily for 8 weeks in a standing position, beginning the day after the removal of drains from the postoperative wound. In addition, all women in the study were required to follow the training strictly according to the instructions and to keep a training diary [30]. In order to assess the quality of the program, the patients were monitored by a physiotherapist during follow-up visits at the Breast Unit.

In group I-IMT, inspiratory muscle training was performed on a Threshold IMT device (Respironics, Inc., a subsidiary of Koninklijke Philips N.V. Murrysville, PA, USA) with a load selected after an initial PI_max_ assessment. In accordance with the available literature, an individual training load level was set, starting at 15% of the PI_max_ load from the first test [31,32]. The maximum training load in the last 8 weeks was 60% PI_max_ (Table 1).

In group II-sham-IMT, inspiratory muscle training was also performed using the Threshold IMT device (Respironics, Inc., a subsidiary of Koninklijke Philips N.V. Murrysville, PA, USA) —15% MIP (Table 2) [22].

After setting an individual training load on the Threshold IMT device, each woman underwent her first training session in the presence of a physiotherapist. The inspiratory phase was characterized by a fast, energetic, short, and diaphragmatic inhalation. Exhalation was slow, calm, and prolonged, and it had to be continued until the residual volume (RV) was reached, as, according to the available literature, each subsequent inhalation should begin at this level [33].

### 2.5. Statistical Analysis

Statistical analysis was performed using STATISTICA PL v.12.0 software. Before conducting comparative analyses, the normality of the distribution of the studied parameters was assessed using the Shapiro–Wilk test. Basic descriptive statistics (mean and standard deviation) were then calculated. Qualitative characteristics describing the participants assigned to the study groups were assessed using a two-sided Fisher’s exact test. Student’s *t*-test for independent samples was used to compare somatic characteristics between the two groups. The U Mann–Whitney test was used for non-normally distributed variables. An analysis of variance (ANOVA) was used for comparisons of more than two groups, and Tukey’s post hoc tests were performed for significant results to determine which groups differed. The level of significance was set at *p* < 0.05 in all analyses.

## 3. Results

In Table 3, descriptive statistics are presented considering the body morphology and clinical characteristics of the patients.

Statistical analysis of the somatic and clinical characteristics of the patients enrolled in the study revealed no significant differences between the study group (Group I-IMT) and the control group (Group II-sham-IMT). Clinically, both groups were homogeneous. All patients were diagnosed with invasive ductal carcinoma. The distribution of tumor stages (IIa and IIb) and menopausal status was similar between the two groups. The majority of patients in both groups remained professionally active (Table 3).

An analysis of descriptive statistics of respiratory system functional parameters was performed.

Table 4 presents the main effects of the studied parameters, considering the group and the study conducted. The first step of the analysis of variance showed statistically significant main effects for all parameters. They are marked in bold in the table. Tukey’s post hoc analysis was applied to these parameters.

FVC values differed at each stage of the study, both in the I-IMT and II-sham-IMT groups, but there were no differences between the groups at individual measurement points. A significant decrease in FVC values was recorded after the procedure in both groups (test 2), and a significant increase 4 weeks after the procedure (before radiotherapy, test 3) was also recorded in both groups. However, in examination 4, i.e., after radiotherapy, a significant increase in FVC was recorded in the I-IMT group and a significant decrease in the II-sham-IMT group. After completion of the entire treatment after approximately 3 weeks (follow-up, examination 5), a significant decrease in FVC was recorded in group I-IMT, but it was significantly higher compared to the examination after surgery, while in group II-sham a significant increase in FVC was recorded, also in relation to the examination after surgery (Table 4, Table 5, Table 6, Table 7 and Table 8).

Forced expiratory volume in one second (FEV_1_) values differed at each stage of the study, both in the I-IMT and II-sham groups, but there were no differences between the groups at individual measurement points. A significant decrease in FEV_1_ was noted after the procedure (study 2) in both groups, and a significant increase was noted four weeks after the procedure (before radiotherapy, study 3) in both groups. However, in study 4, i.e., after radiotherapy, a significant decrease was noted in the II-sham group. After approximately three weeks of treatment (follow-up, study 5), a significant increase in FEV_1_ was noted in both the I-IMT and II-sham-IMT groups, compared to the post-surgery values (Table 4, Table 5, Table 6, Table 7 and Table 8).

Peak expiratory flow (PEF) values differed at each stage of the study, both in the I-IMT and II-sham groups, but there were no differences between the groups at individual measurement points. A significant decrease in PEF values was recorded after the procedure (examination 2) in both groups, and a significant increase 4 weeks after the procedure (before radiotherapy, examination 3) was also observed in both groups. However, in examination 4, i.e., after radiotherapy, a significant decrease was observed in both the I-IMT and II-sham groups. After completion of treatment, approximately 3 weeks later (follow-up, examination 5), a significant increase in PEF was observed in both the I-IMT and II-sham-IMT groups compared to the values at previous measurement points (Table 4, Table 5, Table 6, Table 7 and Table 8).

Spirometric parameters showed similar trends in both groups, but inspiratory muscle strength was the main variable that differed statistically significantly. Maximal inspiratory muscle pressure (PI_max_) values differed at each stage of the study, in both the I-IMT and II-sham-IMT groups. There were no differences between the groups in the first and second examinations, but in the third, fourth, and fifth examinations, significantly higher values were observed in the I-IMT group. A significant decrease in PI_max_ values was noted after the procedure in both groups, and a significant increase after 4 weeks after the procedure (before radiotherapy, examination 3) was noted only in the I-IMT group. In examination 4, i.e., after radiotherapy, a significant increase in PI_max_ was noted in the I-IMT group, while there were no changes in the II-sham-IMT group. After the end of the complete treatment, i.e., after approximately 3 weeks (follow-up, study 5), a significant reduction in PI_max_ was noted in the I-IMT group; however, it was a significantly higher level compared to the examination after the surgery and before the treatment, while in the II-sham-IMT group there were no significant changes. Also, in relation to the examination before and after the procedure, the strength of the inspiratory muscles did not change (Table 4, Table 5, Table 6, Table 7 and Table 8).

The values of maximal expiratory muscle pressure (PE_max_) differed at each stage of the study, both in the I-IMT and II-sham-IMT groups, and between the groups at individual measurement points. A significant decrease in PE_max_ values was recorded after surgery in both groups (study 2), and a significant increase 4 weeks after surgery (before radiotherapy, study 3) was also recorded in both groups. However, in study 4, i.e., after radiotherapy, a significant increase in PE_max_ was recorded in the I-IMT group. After completion of the entire treatment after approximately 3 weeks (follow-up, examination 5), a significant decrease in PE_max_ was recorded in the I-IMT group, but it was significantly higher compared to the examination after surgery, while in the II-sham-IMT group no significant changes were recorded, and in relation to the examination after surgery, it remained at a significantly higher level (Table 4, Table 5, Table 6, Table 7 and Table 8).

Table 9 presents the results of selected descriptive statistics divided into groups and the quality-of-life surveys conducted among patients.

All analyzed areas of women’s quality of life differed significantly before (study 1) and after treatment (study 5), both in the I-IMT and II-sham-IMT groups, while there were no differences between the groups at both measurement points (Table 9, Table 10 and Table 11).

No significant differences were noted in the physical domain after treatment (study 5). In the psychological and social domains, a highly significant reduction was noted after treatment in both groups, and in the environmental domain, a highly significant reduction was noted in the II-sham-IMT group and a significant reduction in the study group. The quality of life assessed by women participating in the study (WHO1) was significantly higher after treatment only in the group additionally undergoing IMT with a load of 60% PI_max_ (Table 9, Table 10 and Table 11). However, it should be emphasized that the quality of life was measured only at the beginning of the study and during the observation period. The results obtained should be interpreted taking into account the short-term nature of the assessment.

## 4. Discussion

Our study results showed a negative effect of the procedure on the assessed parameters of respiratory function and respiratory muscle strength, with a significant increase four weeks after the procedure. However, additional inspiratory muscle training (IMT) with a load of 60% PI_max_ maintained or significantly increased these parameters compared to the sham IMT group. All assessed areas of women’s quality of life deteriorated significantly after treatment. However, the quality of life assessed by the study women (WHO1) was significantly higher after treatment only in women who additionally received IMT. Therefore, it can be concluded that inspiratory muscle training had a positive effect on ventilatory function in women treated for breast cancer, minimizing the negative effects of treatment. Current research indicates that after IMT (inspiratory muscle training), by increasing inspiratory muscle strength, the selective workload of the respiratory system is reduced during spontaneous breathing through the inspiratory muscles. According to Brown et al., this may result in a reduction in net lactic acid production in the muscles [34]. Downey et al. [35] note that this may be due to increased diaphragm strength. Furthermore, Ramirez-Sarmiento et al. also mention an increase in intercostal muscle strength in their study. They emphasize that this mechanism could have led to a reduction in the individual work performed by the inspiratory muscles by delaying the activation of accessory inspiratory muscles. This could have resulted in minimizing lactate formation by inspiratory muscle fibers [36]. Our own studies have shown that, thanks to the use of IMT, the strength of the respiratory muscles in women after cancer treatment significantly improved.

The results of our own research showed a significant reduction in respiratory function and a decrease in respiratory muscle strength, which is consistent with the findings of other authors. Suesady et al. conducted a study assessing the impact of radiotherapy on respiratory function and exercise capacity in patients treated for breast cancer [12]. The study included 37 patients who had completed surgery and radiotherapy for breast cancer. The results presented by the authors clearly indicated a significant reduction in respiratory muscle strength, reduced chest wall mobility, and reduced exercise capacity. In addition, radiotherapy involving supraclavicular lymph nodes was a factor in the development of radiation pneumonitis [12].

Illini et al. demonstrated the effectiveness of introducing respiratory rehabilitation into the therapeutic process during cancer [37]. At the same time, Przedborska et al. showed a significant reduction in the severity of dyspnea thanks to the use of physiotherapy in the treatment of patients [18]. This thesis is confirmed by the results of our own research. The I-IMT group, which underwent inspiratory muscle training 60% PI_max_ during therapy, showed a reduction in respiratory symptoms. The women did not complain of shortness of breath or chest heaviness, and their respiratory parameters improved significantly.

Our findings confirm the conclusions of other authors regarding the improvement of respiratory function through the use of a systematic respiratory rehabilitation program. Odinets et al. conducted a study on the impact of individualized exercise rehabilitation programs on respiratory function in women after mastectomy. The results suggest that individual exercise rehabilitation programs can be considered effective in improving respiratory function in patients after mastectomy [38].

Research on the quality of life of breast cancer patients was evaluated by Costa et al., who surveyed 410 people treated for breast cancer. The results of their research clearly indicate a problem with both the reduced quality of life of patients and the deterioration of their functional status [13]. Comparing the above results with the results of our own research, it can be observed that the theories obtained differ only in minor values. The women surveyed rated their current health as average. Based on the results obtained, it can be concluded that most chronic diseases, and in particular malignant tumors, significantly reduce women’s health. The presented research shows preliminary observations on the supportive potential of IMT in selected individuals who were treated for breast cancer.

A 2022 review of studies by Viñolo-Gil MJ et al. on respiratory physiotherapy intervention strategies for the treatment of breast cancer sequelae indicates some gaps in the use of various respiratory procedures in therapy. Ten studies (six clinical trials, one case study, and three observational studies) conducted at various institutions between 2005 and 2020 were analyzed. Study quality was assessed using the STROBE checklist and the SCED and PEDro scales. The conclusion was that respiratory physiotherapy is not widely used for the treatment of breast cancer sequelae. It improves lung function and exercise tolerance, reduces dyspnea and fatigue, increases chest wall mobility, improves sleep quality and quality of life, and reduces sensitivity to adverse physiological reactions such as nausea, vomiting, and anxiety [39]. The results of our own research also confirm the positive impact of physiotherapy in the examined women from both groups—in all of them, a significant improvement in ventilation parameters was noted 4 weeks after the procedure.

This is also confirmed by other authors, who state that the introduction of oncological physiotherapy allows for the restoration of the psychophysical fitness of women struggling with malignant breast cancer [40]. The results of our own research and their analysis showed that the use of postoperative physiotherapy and the addition of inspiratory muscle training has a beneficial effect on improving the functional status and quality of life of the women studied.

The limitation of this study was the size of the group. The size of the group resulted from the inclusion criteria and the specificity of our population. Larger multicenter studies should be conducted to further investigate the potential role and overall acceptance of this intervention as a physiotherapy tool in selected patients after breast cancer treatment. Another limitation is that the analysis included only patients with HER2-positive breast cancer undergoing neoadjuvant chemotherapy. In future studies, expanding the inclusion criteria to include patients with other biological subtypes of breast cancer, including HER2-negative breast cancer, and with different treatment regimens could yield more diverse results. This approach could enable comparison of the effects of breast-conserving therapy and radiotherapy on lung function and quality of life, depending on the specific tumor type and treatment regimen. The observation period in this study covered the period before surgery, after surgery, during radiotherapy, and one month after its completion, which is a relatively short period in the context of assessing the long-term effects of treatment. This limitation resulted from the study design, which aimed to evaluate the early effects of breast-conserving therapy and radiotherapy, including their impact on respiratory function and quality of life in patients. Therefore, the results should be interpreted as early responses of the body to treatment, and future studies should include a longer observation period to assess the long-term effects of treatment, particularly in the context of changes in respiratory function and quality of life. Further research is needed to identify respiratory physiotherapy techniques necessary to improve specific outcomes in women undergoing surgical and adjuvant therapy [39].

## 5. Conclusions

The individual stages of cancer treatment, including surgery and radiotherapy, have a negative impact on the respiratory function and quality of life in women treated for breast cancer. Spirometric parameters showed similar trends in both groups. In both groups, surgery caused a greater reduction in the mean values of the respiratory function parameters examined than radiotherapy administered at a later stage of treatment. In group II-sham-IMT (sham load 15% PI_max_), a further decrease in the mean values of the respiratory function parameters examined was observed after radiotherapy, which was not observed in group I-IMT with additional inspiratory muscle training at 60% PI_max_. The use of prehabilitation and postoperative physiotherapy reduced the adverse effects of radical breast cancer treatment on respiratory function. The additional use of higher-load inspiratory muscle training enhanced the therapeutic effect of physiotherapy in restoring normal respiratory function. Components of quality of life after treatment remained at a reduced level, with only the subjective assessment of quality of life by women improving, at a higher level in women who underwent inspiratory muscle training. Quality of life was assessed only at the beginning of the study and during follow-up, which means that the observed changes primarily relate to the short-term effects of treatment. Future studies should include a long-term assessment of patients’ quality of life to enable a more comprehensive analysis of its dynamics over time.

## Figures and Tables

**Table 1:** Inspiratory muscle strength training in Group I-IMT.

Training Week	1	2	3	4	5	6	7	8
**Training load** **(cm H_2_O)**	15% PI_max_	20% PI_max_	30% PI_max_	40% PI_max_	50% PI_max_	50% PI_max_	60% PI_max_	60% PI_max_
**Session time (min)**	2 × 5	2 × 8	2 × 11	2 × 11	2 × 13	2 × 13	2 × 15	2 × 15

**Table 2:** Inspiratory muscle strength training in Group II-sham-IMT.

Training Week	1	2	3	4	5	6	7	8
**Training load** **(cm H_2_O)**	constant load 15% PI_max_							
**Session time (min)**	2 × 5	2 × 8	2 × 11	2 × 11	2 × 13	2 × 13	2 × 15	2 × 15

**Table 3:** Somatic and clinical characteristics of the study groups.

Variable	Group I-IMT (n = 13)	Group II-sham-IMT (n = 13)	*p*
	Mean ± SD	Mean ± SD	
Age [years]	52.38 ± 11.39	48.15 ± 10.74	0.3396
Body height [cm]	168.85 ± 7.28	166.62 ± 5.20	0.4294
Body weight [kg]	69.54 ± 6.80	64.38 ± 4.74	0.3776
BMI [kg/m^2^]	25.54 ± 2.50	23.65 ± 1.74	0.4293
Classification of tumor: IIa	7	6	1
IIb	6	7	1
Type of tumor: Invasive ductal carcinoma	13	13	1
Menopause status, n (%) Premenopausal	5	8	0.2743
Postmenopausal	8	5	0.2743
Professional status Active	10	11	1
Inactive	-	-	-
Pensioned	3	2	1

**Table 4:** Characteristics of the respiratory system functional parameters studied in the selected groups and studies.

Variable	Group	Test 1	Test 2	Test 3	Test 4	Test 5
		(Mean *±* SD)	(Mean *±* SD)	(Mean *±* SD)	(Mean *±* SD)	(Mean *±* SD)
**FVC [L]**	**Group I-IMT**	3.34 ± 0.65	2.96 ± 0.6	3.23 ± 0.65	3.45 ± 0.73	3.28 ± 0.66
	**Group II-sham-IMT**	3.69 ± 0.64	3.29 ± 0.60	3.47 ± 0.61	3.41 ± 0.6	3.51 ± 0.61
**FEV_1_ [L]**	**Group I-IMT**	2.71 ± 0.6	2.40 ± 0.55	2.56 ± 0.58	2.58 ± 0.58	2.60 ± 0.58
	**Group II-sham-IMT**	3.02 ± 0.55	2.69 ± 0.51	2.84 ± 0.52	2.79 ± 0.52	2.89 ± 0.5
**PEF [L/s]**	**Group I-IMT**	6.64 ± 0.83	5.87 ± 0.77	6.26 ± 0.79	6.16 ± 0.8	6.34 ± 0.8
	**Group II-sham-IMT**	7.23 ± 0.97	6.43 ± 0.9	6.80 ± 0.92	6.68 ± 0.92	6.87 ± 0.92
**PI_max_ [cm H_2_O]**	**Group I-IMT**	81.08 ± 14.75	72.16 ± 13.46	101.30 ± 26.39	117.94 ± 30.26	110.20 ± 29.45
	**Group II-sham-IMT**	86.99 ± 9.51	76.77 ± 8.65	81.52 ± 9.02	80.28 ± 9.09	82.15 ± 9.56
**PE_max_ [cm H_2_O]**	**Group I-IMT**	122.52 ± 15.48	109.15 ± 14.29	123.79 ± 21.5	131.20 ± 26.73	126.34 ± 22.42
	**Group II-sham-IMT**	129.28 ± 15.43	114.37 ± 14.57	120.85 ± 14.78	118.63 ± 14.56	122.27 ± 14.91

Abb.: FVC—forced vital capacity; FEV_1_—forced expiratory volume in one second; PEF—peak expiratory flow; PI_max_—maximal inspiratory pressure; PE_max_—maximal expiratory pressure.

**Table 5:** Main effects of the examined parameters, considering the group and the study conducted.

Variable	Group		R1—Repeat		R1 × Group	
	F	*p*	F	*p*	F	*p*
**FVC**	1.79	0.1937	213.73	**0.0000**	1.84	0.1277
**FEV_1_**	1.70	0.2043	172.17	**0.0000**	1.25	0.2971
**PEF**	2.62	0.1185	733.11	**0.0000**	1.96	0.1063
**PI_max_**	5.03	**0.0344**	52.31	**0.0000**	50.56	**0.0000**
**PE_max_**	0.05	0.8246	29.23	**0.0000**	13.37	**0.0000**

Abb.: *p* < 0.05 is bolded; FVC—forced vital capacity; FEV_1_—forced expiratory volume in one second; PEF—peak expiratory flow; PI_max_—maximal inspiratory pressure; PE_max_—maximal expiratory pressure.

**Table 6:** Evaluation of the variation of the mean values of the studied parameters; analysis of variance for repeated measurements, Tukey’s test; and probabilities for post hoc tests.

Variable	Probabilities for Post Hoc Tests, Tukey’s Test, *p*-Value				
	Group I-IMT—Group II-sham-IMT				
	Test 1	Test 2	Test 3	Test 4	Test 5
**FVC [L]**	0.1593	0.1823	0.1994	0.2556	0.1862
**FEV_1_ [L]**	0.1738	0.2018	0.2102	0.2593	0.1891
**PEF [L/s]**	0.0952	0.1085	0.1234	0.1429	0.1297
**PI_max_ [cm H_2_O]**	0.4127	0.5222	0.0093	0.0000	0.0004
**PE_max_ [cm H_2_O]**	0.3449	0.4647	0.6790	0.0852	0.5682

Abb.: *p* < 0.05 is bolded; FVC—forced vital capacity; FEV_1_—forced expiratory volume in one second; PEF—peak expiratory flow; PI_max_—maximal inspiratory pressure; PE_max_—maximal expiratory pressure.

**Table 7:** Assessment of the variation in the mean values of the tested parameters in group I-IMT; analysis of variance for repeated measurements, Tukey’s test; and probabilities for post hoc tests.

Variable	Probabilities for Post Hoc Tests, Tukey’s Test, *p*-Value									
	Group I-IMT									
	t1–t2	t1–t3	t1–t4	t1–t5	t2–t3	t2–t4	t2–t5	t3–t4	t3–t5	t4–t5
**FVC [L]**	**0.0000**	**0.0000**	**0.0000**	**0.0000**	**0.0000**	**0.0000**	**0.0000**	0.1733	0.1862	**0.0081**
**FEV_1_ [L]**	**0.0000**	**0.0000**	**0.0000**	**0.0000**	**0.0000**	**0.0000**	**0.0000**	0.1714	**0.0458**	**0.0010**
**PEF [L/s]**	**0.0000**	**0.0000**	**0.0000**	**0.0000**	**0.0000**	**0.0000**	**0.0000**	**0.0000**	**0.0006**	**0.0000**
**PI_max_ [cm H_2_O]**	**0.0016**	**0.0000**	**0.0000**	**0.0000**	**0.0000**	**0.0000**	**0.0000**	**0.0000**	**0.0017**	**0.0059**
**PE_max_ [cm H_2_O]**	**0.0000**	0.5521	**0.0001**	0.0772	**0.0000**	**0.0000**	**0.0000**	**0.0008**	0.2371	**0.0253**

Abb.: *p* < 0.05 is bolded; FVC–forced vital capacity; FEV_1_—forced expiratory volume in one second; PEF—peak expiratory flow; PI_max_—maximal inspiratory pressure; PE_max_—maximal expiratory pressure.

**Table 8:** Assessment of the variation in the mean values of the tested parameters in group II-sham-IMT; analysis of variance for repeated measurements, Tukey’s test; and probabilities for post hoc tests.

Variable	Probabilities for Post Hoc Tests, Tukey’s Test, *p*-Value									
	Group II-sham-IMT									
	t1–t2	t1–t3	t1–t4	t1–t5	t2–t3	t2–t4	t2–t5	t3–t4	t3–t5	t4–t5
**FVC [L]**	**0.0000**	**0.0000**	**0.0000**	**0.0000**	**0.0000**	**0.0000**	**0.0000**	**0.0012**	0.0665	**0.0000**
**FEV_1_ [L]**	**0.0000**	**0.0000**	**0.0000**	**0.0000**	**0.0000**	**0.0000**	**0.0000**	**0.0037**	**0.0062**	**0.0000**
**PEF [L/s]**	**0.0000**	**0.0000**	**0.0000**	**0.0000**	**0.0000**	**0.0000**	**0.0000**	**0.0000**	**0.0024**	**0.0000**
**PI_max_ [cm H_2_O]**	**0.0003**	**0.0495**	**0.0164**	0.0812	0.0870	0.2050	0.0534	0.6514	0.8212	0.4982
**PE_max_ [cm H_2_O]**	**0.0000**	**0.0002**	**0.0000**	**0.0015**	**0.0032**	**0.0496**	**0.0004**	0.3020	0.5077	0.0919

Abb.: *p* < 0.05 is bolded; FVC—forced vital capacity; FEV_1_—forced expiratory volume in one second; PEF—peak expiratory flow; PI_max_—maximal inspiratory pressure; PE_max_—maximal expiratory pressure.

**Table 9:** Characteristics of quality-of-life components in groups and tests.

Variable	Group	Before Treatment	After Treatment (Follow-Up)
		Mean ± SD	Mean ± SD
**Domain 1** **(Physical QOL)**	group I-IMT	11.69 ± 1.35	10.95 ± 1.09
	group II-sham-IMT	10.99 ± 0.94	11.16 ± 0.95
**Domain 2 (Psychological QOL)**	group I-IMT	14.56 ± 1.49	10.10 ± 1.49
	group II-sham-IMT	14.41 ± 1.29	10.51 ± 1.02
**Domain 3** **(Social Relationships QOL)**	group I-IMT	14.97 ± 1.11	11.59 ± 2.14
	group II-sham-IMT	15.08 ± 1.48	11.79 ± 1.71
**Domain 4** **(Environmental QOL)**	group I-IMT	13.69 ± 0.52	12.77 ± 1.13
	group II-sham-IMT	13.85 ± 0.77	12.69 ± 0.69
**WHO1**	group I-IMT	3.71 ± 0.75	4.35 ± 0.49
	group II-sham-IMT	4.07 ± 0.50	3.96 ± 0.70
**WHO2**	group I-IMT	3.40 ± 0.89	3.38 ± 0.91
	group II-sham-IMT	3.63 ± 0.84	3.22 ± 0.93

Abb.: WHO1, individual overall perception of quality of life; WHO2, individual overall perception of health.

**Table 10:** Assessment of the variation in the mean values of the parameters studied; analysis of variance for repeated measurements, main effects.

Variable	Group		R1—Repeat		R1 × Group	
	F	*p*	F	*p*	F	*p*
**Domain 1**	0.55	0.4658	1.04	0.3173	2.72	0.1120
**Domain 2**	0.15	0.7049	107.35	**0.0000**	0.49	0.4911
**Domain 3**	0.15	0.7000	42.18	**0.0000**	0.01	0.9212
**Domain 4**	0.02	0.8833	31.87	**0.0000**	0.39	0.5364
**WHO1**	1.18	0.281	7.92	**0.009**	0.91	0.343
**WHO2**	0.34	0.562	0.77	0.382	0.29	0.592

Abb.: *p* < 0.05 is bolded; WHO1, individual overall perception of quality of life; WHO2, individual overall perception of health.

**Table 11:** Significance of differences in quality of life before and after treatment in the I-IMT and II-sham-IMT groups.

Variable	Probabilities for Post Hoc Tests, Tukey’s Test, *p*-Value			
	Before Treatment—After Treatment (Follow-Up)		Group I-IMT—Group II-sham-IMT	
	Group I-IMT	Group II-sham-IMT	Before Treatment	After Treatment (Follow-Up)
**Domain 1**	0.0711	0.6607	0.1084	0.6115
**Domain 2**	0.0000 *	0.0000 *	0.7704	0.4377
**Domain 3**	0.0001 *	0.0001 *	0.8748	0.7528
**Domain 4**	0.0016 *	0.0002 *	0.6309	0.8100
**WHO1**	0.006 *	0.074	0.5609	0.8711
**WHO2**	0.13215	0.1685	0.9307	0.2111

Abb.: * *p* < 0.05 is bolded; WHO1, individual overall perception of quality of life; WHO2, individual overall perception of health.

## Data Availability

The data presented in this study are available upon request from the corresponding author.

## Abbreviations

The following abbreviations are used in this manuscript:

BMI	Body mass index
FVC	Forced vital capacity
FEV_1_	Forced vital capacity
PEF	Peak expiratory flow
PI_max_	Maximal inspiratory pressure
PE_max_	Maximal expiratory pressure
IMT	Inspiratory muscle training
WHOQOL	Word Health Organization Quality of Life
WHO1	Individual overall perception of quality of life
WHO2	Individual overall perception of health
QOL	Quality of life
